# The effect of pelvic floor muscle exercise, progressive muscle relaxation exercise, and their combined application on comfort in women with stress urinary incontinence: a four-arm randomized controlled trial

**DOI:** 10.3389/fmed.2026.1967374

**Published:** 2026-10-20

**Authors:** Ahmet Şanlı, Gamze Göke Arslan, Demet Çelik, Burak Demir

**Affiliations:** 1 Department of Urology, Faculty of Medicine, Karamanoğlu Mehmetbey University, Karaman, Türkiye; 2 Department of Nursing, Faculty of Health Sciences, Karamanoğlu Mehmetbey University, Karaman, Türkiye; 3 Department of Physical Therapy and Rehabilitation, Karaman Training and Research Hospital, Karaman, Türkiye

**Keywords:** comfort, pelvic floor muscle exercise, progressive muscle relaxation exercise, randomized controlled trial, stress urinary incontinence

## Abstract

**Background:**

Stress urinary incontinence (SUI) negatively affects women's physical, psychological, and social well-being and reduces comfort. Although pelvic floor muscle exercise (PFME) is the first-line conservative treatment, the additional contribution of progressive muscle relaxation exercise (PMR) to patient comfort remains unclear. This study aimed to evaluate the effects of PFME, PMR, and their combined application on comfort levels in women with SUI.

**Methods:**

This four-arm randomized controlled trial included 80 women diagnosed with SUI who were randomly assigned to the PFME group (*n* = 20), PMR group (*n* = 20), PFME + PMR group (*n* = 20), or control group (*n* = 20). Data were collected using a Descriptive Information Form and the Urinary Incontinence Frequency and Comfort Questionnaire (UIFCQ). Participants in the intervention groups performed the assigned exercise program for six weeks. Comfort was assessed at baseline and at Weeks 2, 4, and 6 through telephone interviews. Data were analyzed using descriptive statistics and mixed-design analysis of variance (Mixed ANOVA). A *p*-value of <0.05 was considered statistically significant.

**Results:**

Baseline comfort scores did not differ significantly among the groups (*p* > 0.05). Significant effects of time and the group×time interaction were observed (*p* < 0.001). Participants in the PFME group demonstrated significant improvements in comfort beginning at Week 2, which were maintained throughout the study. The PMR group showed gradual improvements in comfort across the follow-up period. In the combined PFME + PMR group, significant improvements became evident at the third and fourth assessments. Although slight changes were observed in the control group over time, the trajectories of improvement in all intervention groups were significantly greater than those in the control group.

**Conclusions:**

PFME, PMR, and their combined application improved comfort over time in women with stress urinary incontinence. These findings suggest that structured conservative interventions, particularly the integration of PMR into pelvic floor rehabilitation programs, may enhance patient comfort and support comprehensive SUI management. Further multicenter randomized controlled trials with larger sample sizes and longer follow-up periods are warranted.

**Clinical trial registration:**

ClinicalTrials.gov, Identifier: NCT07762391, https://clinicaltrials.gov/study/NCT07762391

## Graphical Abstract

**Figure:**
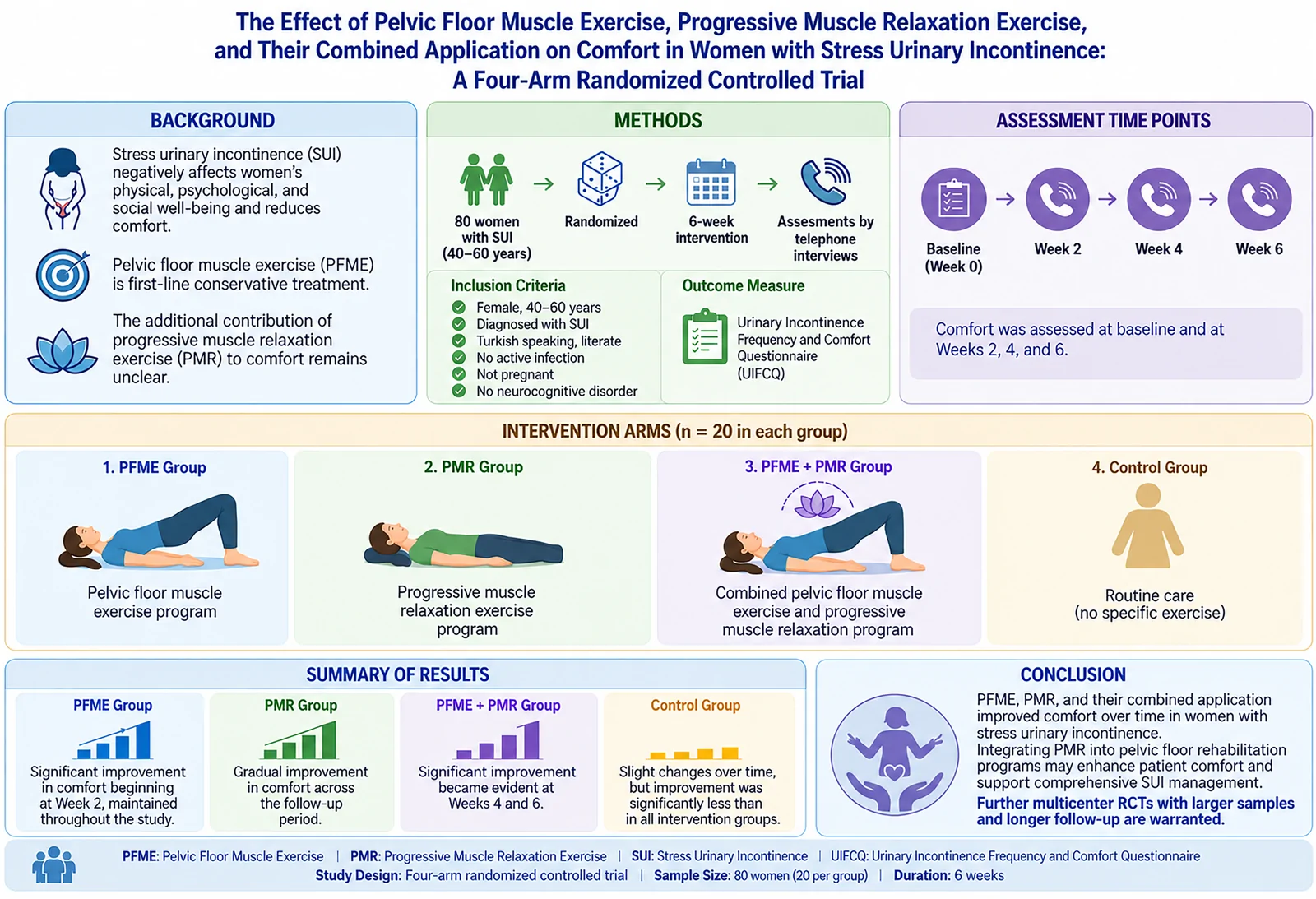
Overview of the four–arm randomized controlled trial evaluating the effects of pelvic floor muscle exercise (PFME), progressive muscle relaxation (PMR), and their combined application on comfort in women with stress urinary incontinence over a six–week intervention period.

## 1 Introduction

Urinary incontinence (UI) is defined by the International Continence Society (ICS) as any complaint of involuntary leakage of urine (1). Beyond being a urological disorder, UI is recognized as a major public health concern with substantial physical, psychological, social, and economic consequences. Although it can occur at any age, UI is approximately twice as common in women as in men, and its prevalence increases significantly with advancing age. This higher prevalence is largely attributable to pregnancy, vaginal childbirth, menopause, pelvic floor muscle weakness, and hormonal changes, all of which contribute to pelvic floor dysfunction and urinary symptoms (2, 3).

Epidemiological studies indicate that approximately 25%–45% of women worldwide experience UI at some point during their lifetime, with the prevalence exceeding 50% among women aged 60 years and older (2, 4, 5). However, the true prevalence is likely underestimated because many women do not seek medical care due to embarrassment, the belief that urinary leakage is a normal consequence of aging, or the misconception that effective treatment is unavailable (2, 4). According to the ICS, UI is classified into stress urinary incontinence (SUI), urgency urinary incontinence (UUI), mixed urinary incontinence (MUI), and other less common subtypes. Among women, SUI is the most prevalent form and is defined as the involuntary leakage of urine during activities that increase intra-abdominal pressure, such as coughing, sneezing, laughing, running, or lifting heavy objects. In contrast, MUI is characterized by the coexistence of symptoms of both stress and urgency urinary incontinence (5).

Several risk factors have been identified for the development of urinary incontinence in women, including advancing age, obesity, parity, vaginal delivery, menopause, chronic cough, constipation, previous pelvic surgery, connective tissue disorders, and genetic predisposition. Pregnancy and vaginal childbirth may result in injury to the pelvic floor muscles, connective tissues, and pudendal nerve, thereby compromising the continence mechanism. In addition, estrogen deficiency during menopause contributes to urogenital atrophy, further increasing the risk of urinary incontinence (6).

Although urinary incontinence is not a life-threatening condition, it has a profound negative impact on women's quality of life. Fear of urine leakage may lead women to withdraw from social activities, limit physical activity, and experience difficulties in performing daily living activities. Furthermore, urinary incontinence is frequently associated with psychological distress, including depression, anxiety, embarrassment, reduced self-esteem, sexual dysfunction, and sleep disturbances. It also imposes a considerable economic burden through increased healthcare expenditures, the use of continence products, and productivity loss (7). These consequences indicate that the impact of SUI extends beyond urinary symptoms and may affect women's physical, psychological, and social experiences.

Comfort is a multidimensional concept encompassing physical, psychospiritual, sociocultural, and environmental well-being, as described in Kolcaba's Comfort Theory (8). In women with urinary incontinence, comfort may be compromised by persistent urine leakage, embarrassment, anxiety, limitations in daily activities, and reduced social participation (9, 10). According to Kolcaba's Comfort Theory, comfort is not limited to the absence of symptoms; rather, it refers to a holistic state resulting from the satisfaction of individuals' needs for relief, ease, and transcendence across physical, psychospiritual, sociocultural, and environmental contexts (8). Therefore, comfort may be a clinically meaningful patient-centered outcome in women with SUI because it can capture the broader consequences of urinary incontinence, including perceived control, physical ease, psychological well-being, and the ability to participate in daily and social activities. Recent randomized evidence has also demonstrated that a Comfort Theory-based intervention can improve comfort and urinary incontinence-related outcomes in women with SUI, supporting the clinical relevance of comfort as an outcome in this population (11).

Importantly, comfort should be conceptually distinguished from symptom burden and quality of life. Symptom burden primarily reflects the frequency, severity, and perceived impact of urinary symptoms, whereas quality of life describes the extent to which urinary incontinence affects broader aspects of physical, psychological, and social functioning. In contrast, comfort represents a multidimensional state of perceived relief and ease encompassing physical, psychospiritual, sociocultural, and environmental contexts (12). Therefore, comfort should not be regarded simply as another measure of quality of life; rather, it may provide complementary information about how women experience and respond to SUI and how nursing interventions influence their overall sense of well-being. This distinction is particularly relevant to nursing research because an intervention may improve a woman's perceived comfort through reductions in fear, tension, or perceived loss of control even when changes in urinary symptom severity or quality of life are not proportional.

The pelvic floor consists of a complex network of muscles and connective tissues that provide structural support to the bladder, urethra, uterus, and rectum. Adequate muscle strength, endurance, and neuromuscular coordination are essential for maintaining urinary continence. Weakness of the pelvic floor muscles, impaired neuromuscular function, or damage to the supporting connective tissues may disrupt the continence mechanism and contribute to the development of urinary incontinence. Therefore, restoring pelvic floor muscle function constitutes the cornerstone of conservative management for women with urinary incontinence (13).

International clinical guidelines recommend pelvic floor muscle exercise (PFME) as the first-line conservative treatment for women with stress urinary incontinence (SUI) and mixed urinary incontinence (MUI). The primary objective of PFME is to improve pelvic floor muscle strength, endurance, coordination, and voluntary muscle contraction, thereby enhancing urethral closure pressure and maintaining urinary continence. Evidence from randomized controlled trials has consistently demonstrated that regular and correctly performed PFME reduces the frequency of urinary leakage, increases pelvic floor muscle strength, and improves quality of life in women with urinary incontinence (14, 15). Beyond these effects on urinary symptoms, improved continence control may reduce concerns related to accidental leakage, increase perceived control over bodily functions, and facilitate participation in daily and social activities. Through these pathways, PFME may contribute not only to symptom improvement but also to greater physical, psychospiritual, and sociocultural comfort. This proposed relationship is supported by previous research showing that pelvic floor re-education can improve comfort in women with stress urinary incontinence (12).

However, the effectiveness of PFME depends on more than muscle strengthening alone. Treatment success is strongly influenced by adherence to the exercise program, patient motivation, the ability to correctly activate the pelvic floor muscles, anxiety levels, and long-term compliance. Psychological factors, particularly anxiety and chronic stress, may induce excessive pelvic floor muscle tension, impair neuromuscular coordination, and increase symptom perception, potentially reducing the effectiveness of conservative treatment (16, 17).

Progressive muscle relaxation exercise (PMR), originally developed by Edmund Jacobson, is a relaxation technique based on the sequential contraction and relaxation of specific muscle groups. By regulating autonomic nervous system activity, PMR reduces sympathetic nervous system activation, enhances parasympathetic activity, alleviates muscle tension, and promotes both physical and psychological relaxation. Numerous studies have demonstrated its effectiveness in the management of chronic pain, cancer-related symptoms, hypertension, anxiety, sleep disturbances, and stress. Furthermore, relaxation techniques have been suggested to improve pelvic floor muscle coordination, reduce excessive muscle tension, and enhance adherence to conservative treatment programs in women with urinary incontinence (18). Although PMR is not primarily a continence-strengthening intervention, its potential relevance to comfort lies in its ability to reduce physical and psychophysiological tension and promote relaxation. Thus, PMR may be particularly relevant to the physical and psychospiritual dimensions of comfort.

While PFME primarily aims to improve muscle strength and functional support of the pelvic floor, PMR focuses on regulating muscle tone, reducing stress, and promoting psychological well-being. Accordingly, these interventions may influence comfort through partially distinct but complementary pathways. PFME primarily targets the physical and functional dimensions of SUI by improving pelvic floor muscle function and continence control, whereas PMR may address physical tension and psychophysiological distress and thereby promote relaxation and ease. From the perspective of Comfort Theory, the combination of PFME and PMR may therefore address both functional and psychophysiological aspects of SUI simultaneously. This theoretical complementarity provides a rationale for examining whether the combined intervention produces greater improvements in comfort than either intervention alone or standard care. Although numerous randomized controlled trials have demonstrated the effectiveness of PFME, evidence investigating the combined use of PFME and PMR, particularly with respect to comfort as an outcome, remains limited (7, 14). Previous research has predominantly focused on outcomes such as urinary leakage frequency, pad test results, pelvic floor muscle strength, symptom severity, and quality of life, whereas comfort has received comparatively limited attention as an independent patient-centered outcome. Although previous research has demonstrated an effect of pelvic floor re-education on comfort in women with SUI (12), and recent evidence has further supported the relevance of comfort as an outcome in this population (11), evidence specifically comparing the effects of PFME, PMR, and their combined application on comfort remains limited. This represents an important evidence gap because the relative and combined contributions of a strengthening-based intervention and a relaxation-based intervention to comfort have not been adequately established. Consequently, investigating the effects of conservative interventions on comfort may provide valuable evidence for optimizing clinical practice and supporting evidence-based management of stress urinary incontinence.

Accordingly, this four-arm randomized controlled trial was designed to compare the effects of PFME, PMR, their combined application, and standard care on comfort levels in women with stress urinary incontinence. We hypothesized that the combined intervention would provide greater improvements in comfort than either intervention alone or standard care. By evaluating comfort as the primary outcome, this study aims to extend the evidence beyond urinary symptom reduction and quality-of-life outcomes and to provide a more comprehensive understanding of how conservative interventions influence the multidimensional well-being of women with SUI.

The study was designed to test the following hypotheses:

H1: Women with stress urinary incontinence who receive pelvic floor muscle exercise will demonstrate significantly higher post-intervention comfort levels than those receiving standard care.

H2: Women with stress urinary incontinence who receive progressive muscle relaxation exercise will demonstrate significantly higher post-intervention comfort levels than those receiving standard care.

H3: Women with stress urinary incontinence who receive the combined PFME and PMR intervention will demonstrate significantly higher post-intervention comfort levels than those receiving standard care.

## 2 Materials and methods

### 2.1 Study design

This study was designed as a four-arm, parallel-group, randomized controlled trial and was conducted in accordance with the Consolidated Standards of Reporting Trials (CONSORT) 2010 Statement (19).

### 2.2 Study setting and participants

The study was conducted at the Urology Outpatient Clinic of Karaman Training and Research Hospital, Karaman, Türkiye, between 1 June 2025 and 1 May 2026.

The study population consisted of women presenting to the outpatient clinic with symptoms of stress urinary incontinence (SUI) during the study period. The study sample comprised women who met the eligibility criteria, received detailed information about the study, provided written informed consent, and voluntarily agreed to participate.

The inclusion criteria were as follows: (1) female sex; (2) a clinical diagnosis of stress urinary incontinence; (3) age between 40 and 60 years; (4) ability to communicate in Turkish; (5) literacy; (6) access to the internet; (7) regular use of a smartphone; (8) absence of active vaginal or urinary tract infection; (9) not being pregnant; and (10) no physician-diagnosed neurological or cognitive disorder.

The exclusion criteria were previous participation in PFME or PMR training programs, failure to meet the eligibility criteria, and withdrawal from the study at any stage after enrollment.

### 2.3 Sample size calculation

The sample size was determined using G*Power version 3.1 (20). An *a priori* power analysis based on data obtained from the first 10 participants enrolled in the pilot study indicated that a minimum of 20 participants per group was required to provide adequate statistical power to detect significant differences among the four study groups. Thus, the minimum required sample size was 80 participants (20 per group). To account for potential attrition and participant loss during the follow-up period, additional participants were recruited, resulting in a total of 96 women being randomized to the four study groups.

### 2.4 Randomization and allocation

Participants were allocated to one of four study groups: the PFME group, the PMR group, the combined PFME + PMR group, or the control group. To minimize cross-contamination between intervention groups in the outpatient setting, a day-based allocation procedure was employed. Clinic days were randomly assigned to the four study groups, and eligible participants were allocated to the group corresponding to the day of their clinic visit.

Four opaque labels representing the clinic days (Monday, Tuesday, Wednesday, and Thursday) were placed in a sealed opaque bag and randomly drawn by an independent researcher not involved in participant recruitment or intervention delivery. The drawn order determined the group assignment for each specific weekday (e.g., first drawn day assigned to PFME, second to PMR, third to combined PFME + PMR, and fourth to control).

All eligible women attending the outpatient clinic on a specific day who provided written informed consent were assigned to that day's designated group. While true block or simple individual randomization was not implemented due to practical contamination risks, allocation of days was managed independently to maintain concealment prior to day assignment.

### 2.5 Blinding

Because of the nature of the interventions, blinding of participants and the rehabilitation specialist who provided the exercise training was not feasible. However, all participants completed the same standardized outcome measure at each assessment time point, and statistical analyses were performed using coded group assignments.

### 2.6 Data collection instruments

#### 2.6.1 Personal information form

A structured Personal Information Form was developed by the researchers based on the relevant literature to obtain participants' sociodemographic and clinical characteristics. The questionnaire included items related to age, weight, height, educational level, income status, health-related characteristics, awareness of PFME and PMR, and urinary incontinence-related variables.

#### 2.6.2 Urinary incontinence and frequency comfort questionnaire (UIFCQ)

Comfort was assessed using the Urinary Incontinence and Frequency Comfort Questionnaire (UIFCQ), which was developed by Dowd et al. based on Kolcaba's General Comfort Theory (8). The Turkish validity and reliability study was conducted by Zengin (10).

The questionnaire consists of 28 items evaluating four dimensions of comfort (physical, psychospiritual, sociocultural, and environmental) across three comfort states (relief, ease, and transcendence). Items are rated on a six-point Likert scale ranging from 1 (“strongly disagree”) to 6 (“strongly agree”). Twelve negatively worded items are reverse scored. Total scores range from 28 to 168, with higher scores indicating greater comfort. In the statistical analysis and data tables of the present study, scores are presented as item-mean values (total score divided by 28, ranging from 1 to 6) to allow a standardized interpretation across comfort dimensions. The Cronbach's alpha coefficient was reported as 0.77 in the Turkish validation study, whereas the internal consistency coefficient in the present study was 0.83 (10).

### 2.7 Intervention

All intervention programs were delivered individually by a specialist in Physical Medicine and Rehabilitation using standardized training protocols. Participants in the intervention groups were instructed to record their daily exercise practice using exercise follow-up forms throughout the six-week intervention period. Adherence was monitored through participant self-report, supported by weekly telephone follow-up and text message reminders from nurse academics. No objective monitoring device or direct observation was used to verify home-based exercise performance.

#### 2.7.1 Pelvic floor muscle exercise group

Participants allocated to the PFME group received standardized pelvic floor muscle training. During the initial session, the anatomy and function of the pelvic floor muscles were explained, and participants were instructed on correct voluntary contraction techniques. Exercises were initially performed in the supine position with the knees flexed. Participants were instructed to contract the pelvic floor muscles for 3–4 s, gradually increasing the contraction time to 10 s as tolerated, followed by an 8–10 s relaxation period. They were instructed to perform at least 10 sets per day, each consisting of 10 contraction-relaxation cycles. Correct breathing techniques were emphasized, and contraction of the abdominal, gluteal, and thigh muscles was discouraged. Participants were encouraged to continue the exercises in different body positions during daily activities. Participants recorded their daily PFME practice on exercise follow-up forms. Weekly telephone follow-up and text message reminders were used to reinforce adherence and address difficulties encountered during home practice. Because adherence was based on participant self-report, the actual frequency and quality of PFME performance could not be objectively verified.

#### 2.7.2 Progressive muscle relaxation exercise group

Participants in the PMR group received standardized relaxation training based on Jacobson's progressive muscle relaxation technique. Following diaphragmatic breathing exercises, major muscle groups, including the feet, legs, hips, abdomen, hands, arms, shoulders, neck, and facial muscles, were sequentially contracted for approximately 5–10 s and then relaxed for 10–20 s. Participants were instructed to perform the relaxation exercises once daily for approximately 20–30 min throughout the six-week intervention period. Participants recorded each daily PMR session on exercise follow-up forms. Weekly telephone follow-up and text message reminders were used to reinforce adherence and clarify the PMR procedure when necessary. As PMR was performed independently at home, the actual duration and completeness of each 20–30-minute session could not be objectively verified. Therefore, adherence to the prescribed daily PMR duration may have varied among participants and was based on self-reported practice.

#### 2.7.3 Combined PFME + PMR group

Participants assigned to the combined intervention group received both PFME and PMR training during the same educational session. They were instructed to perform PFME according to the standard protocol and PMR once daily for approximately 20–30 min. Written educational materials and exercise videos were provided to support home-based practice. Participants recorded their daily PFME and PMR practice on exercise follow-up forms. Weekly telephone follow-up and text message reminders were used to reinforce adherence to both components of the intervention. Because both interventions were performed independently at home, adherence was based on participant self-report, and the actual duration and quality of home-based PFME and PMR practice could not be objectively verified.

#### 2.7.4 Control group

Participants in the control group received routine outpatient care only and did not receive structured exercise training during the study period.

### 2.8 Data collection procedure

Baseline data were collected through face-to-face interviews conducted in a private consultation room within the outpatient clinic. Following confirmation of the diagnosis by a urologist and completion of baseline assessments, participants in the intervention groups received individualized exercise training from a Physical Medicine and Rehabilitation specialist. The initial training session lasted approximately 30–40 min and included practical demonstrations, educational videos, and written home exercise instructions.

Participants were instructed to perform their assigned exercises regularly for six weeks. To enhance adherence, participants in the intervention groups received weekly telephone follow-up and text message reminders from nurse academics. Participants recorded their daily exercise practice on exercise follow-up forms, which were reviewed during the weekly telephone follow-up. Adherence was assessed exclusively through participant self-report; no objective monitoring device, direct observation, or other objective measure was used to verify home-based exercise performance. Therefore, the reported adherence may not fully reflect the actual frequency, duration, or quality of exercise performance.

The UIFCQ was administered at baseline and repeated at Weeks 2, 4, and 6 via telephone interviews to evaluate changes in comfort over time.

Participant screening, eligibility assessment, enrollment, randomization, follow-up, attrition, and analysis were reported in accordance with the CONSORT 2010 Statement (19), and the participant flow diagram is presented in Figure 1.

**Figure 1:**
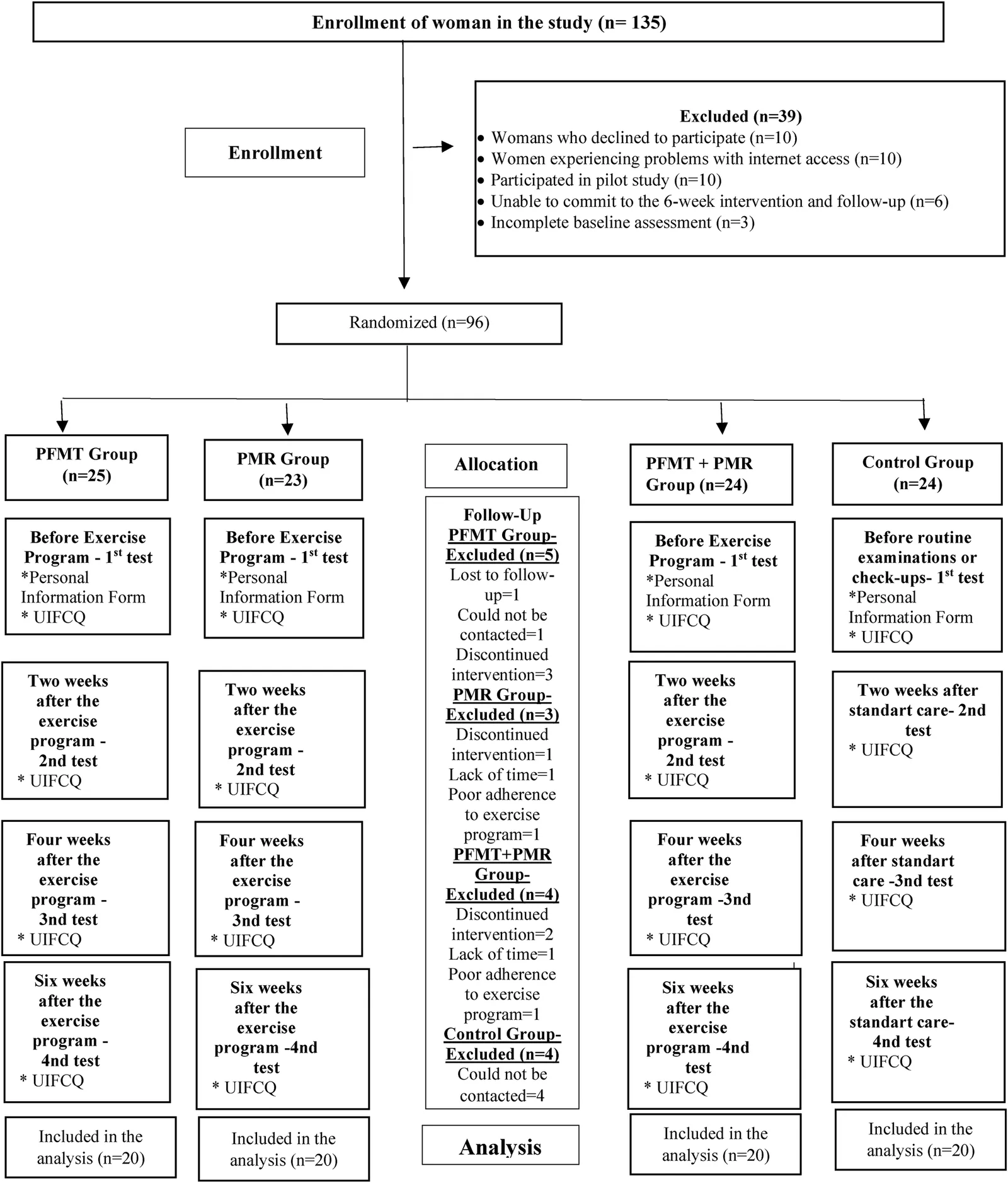
CONSORT flowchart.

### 2.9 Ethical considerations

Ethical approval for this study was obtained from the Ethics Committee of Karamanoğlu Mehmetbey University Faculty of Medicine (approval date: 24 July 2024; approval No. E-11095095-050.04-201209). Written institutional permission was also obtained from Karaman Training and Research Hospital prior to the commencement of the study. Permission to use the Turkish version of the UIFCQ was obtained via e-mail from the author who conducted its Turkish validity and reliability study. The study was conducted in accordance with the ethical principles of the Declaration of Helsinki. Written informed consent was obtained from all participants before enrollment. Participants were informed that their participation was entirely voluntary and that they could withdraw from the study at any time without affecting the healthcare services they received. All personal information and study data were treated as confidential and were used exclusively for scientific research purposes.

### 2.10 Statistical analysis

Statistical analyses were performed using IBM SPSS Statistics for Windows, Version 25.0 (IBM Corp., Armonk, NY, USA). Primary analyses were conducted on a Per-Protocol (PP) basis, including only participants who fully adhered to the intervention protocol and completed all follow-up assessments (*n* = 20 per group, total *N* = 80). Dropouts and non-compliant participants were excluded from the final analysis. Baseline demographic characteristics were compared among the study groups using the chi-square test or Fisher's exact test for categorical variables, and one-way Analysis of Variance (ANOVA) for continuous variables. Data normality was assessed based on skewness and kurtosis values, with values between −1.5 and +1.5 considered indicative of a normal distribution (21). Since the normality assumption was satisfied, parametric tests were applied.

Changes in comfort scores over time (baseline, Weeks 2, 4, and 6) among the four study groups (PFME, PMRE, PFME + PMRE, and control) were analyzed using mixed-design analysis of variance (Mixed ANOVA). Group was specified as the between-subjects factor and time as the within-subjects factor. The main effects of group and time and the group×time interaction were examined. Mauchly's test of sphericity was used to assess the sphericity assumption, and the Greenhouse–Geisser correction was applied when the assumption of sphericity was violated. When a significant group×time interaction was detected, follow-up simple-effects analyses were conducted to examine within-group changes across measurement times. Bonferroni adjustment was applied to control for multiple comparisons. Bonferroni-adjusted *post hoc* multiple comparisons were performed to identify significant main and interaction effects. A two-sided *p*-value of <0.05 was considered statistically significant.

## 3 Results

Table 1 presents the baseline descriptive characteristics of the participants in the four study groups. No statistically significant differences were found among the groups in terms of educational level, income status, chronic disease status, regular medication use, having heard of PFME or PMR, frequency of urinary leakage, age, weight, or height (all *p* > 0.05). These findings indicate that the groups were comparable at baseline before the intervention.

**Table 1:** Comparison of the baseline descriptive characteristics of the study groups.

Descriptive Characteristics	PFME (*n* = 20)	PMR (*n* = 20)	PFME+PMR (*n* = 20)	Control (*n* = 20)	Test	p
Education level						
Literate	2 (10.0)	2 (10.0)	0 (0.0)	3 (15.0)		
Primary school	6 (30.0)	8 (40.0)	7 (35.0)	7 (35.0)		
Secondary school	4 (20.0)	3 (15.0)	3 (15.0)	2 (10.0)	*χ*^2^ = 4.476	0.973
High school	3 (15.0)	2 (10.0)	4 (20.0)	3 (15.0)		
University	5 (25.0)	5 (25.0)	6 (30.0)	5 (25.0)		
Income status						
Income less than expenses	5 (25.0)	6 (30.0)	6 (30.0)	9 (45.0)	χ^2^ = 2.051	0.562
Income equal to expenses	15 (75.0)	14 (70.0)	14 (70.0)	11 (55.0)		
Chronic disease						
Yes	6 (30.0)	7 (35.0)	6 (30.0)	8 (40.0)	χ^2^ = 0.615	0.893
No	14 (70.0)	13 (65.0)	14 (70.0)	12 (60.0)		
Regular medication use						
Yes	6 (30.0)	7 (35.0)	6 (30.0)	7 (35.0)	χ^2^ = 0.228	0.973
No	14 (70.0)	13 (65.0)	14 (70.0)	13 (65.0)		
Having heard of PFME						
Yes	0 (0.0)	1 (5.0)	3 (15.0)	1 (5.0)	χ^2^ = 4.053	0.256
No	20 (100.0)	19 (95.0)	17 (85.0)	19 (95.0)		
Having heard of PMR						
Yes	0 (0.0)	1 (5.0)	2 (10.0)	0 (0.0)	χ^2^ = 3.810	0.283
No	20 (100.0)	19 (95.0)	18 (90.0)	20 (100.0)		
Frequency of urinary leakage						
Once daily	2 (10.0)	1 (5.0)	1 (5.0)	2 (10.0)		
2–4 times daily	6 (30.0)	6 (30.0)	6 (30.0)	6 (30.0)		
Once weekly	7 (35.0)	6 (30.0)	7 (35.0)	7 (35.0)	χ^2^ = 1.968	0.999
2–4 times weekly	3 (15.0)	5 (25.0)	3 (15.0)	3 (15.0)		
Once monthly or less	2 (10.0)	2 (10.0)	3 (15.0)	2 (10.0)		
Age (mean ± SD)	49.50 ± 7.83	53.25 ± 7.79	52.25 ± 7.97	51.50 ± 7.97	F = 0.810	0.492
Weight (mean ± SD)	68.35 ± 10.23	67.15 ± 11.52	65.20 ± 9.85	66.85 ± 11.38	F = 0.291	0.832
Height (mean ± SD)	164.15 ± 5.09	164.55 ± 3.46	164.00 ± 3.46	164.65 ± 3.83	F = 0.121	0.948

PFME, pelvic floor muscle exercise; PMR, progressive muscle relaxation exercise; SD, standard deviation; χ^2^, chi-square test; F, one-way analysis of variance (ANOVA).

As shown in Table 2, a mixed-design analysis of variance (Mixed ANOVA) with four study groups and four repeated measurements was conducted to examine changes in Urinary Incontinence and Frequency Comfort Questionnaire (UIFCQ) scores over time. Prior to the analysis, the assumptions of the model were evaluated. Levene's test indicated homogeneity of error variances across groups at each assessment time point (*p* > 0.05). However, Mauchly's test showed that the assumption of sphericity was violated [W = 0.472, *χ*^2^(5)= 56.030, *p* < 0.001]. Therefore, Greenhouse–Geisser corrections were applied to all within-subject analyses.

**Table 2:** Distribution of urinary incontinence and frequency comfort questionnaire (UIFCQ) scores according to study group and assessment time points and results of the mixed-design analysis of variance (mixed ANOVA).

Study Group	Baseline	Week 2	Week 4	Week 6
	Mean ± SD	Mean ± SD	Mean ± SD	Mean ± SD
PFME (*n* = 20)	4.54 ± 0.41	4.60 ± 0.40	4.60 ± 0.40	4.61 ± 0.40
PMR (*n* = 20)	4.46 ± 0.35	4.61 ± 0.39	4.65 ± 0.40	4.66 ± 0.38
PFME + PMR (*n* = 20)	4.61 ± 0.43	4.65 ± 0.44	4.66 ± 0.44	4.68 ± 0.41
Control (*n* = 20)	4.82 ± 0.39	4.76 ± 0.37	4.74 ± 0.36	4.76 ± 0.37

Effect	**Statistical Result**
Group	*F* = 0.891; *p* = 0.450; partial *η*^2^ = 0.034
Time	*F* = 43.611; *p* < 0.001; partial *η*^2^ = 0.365
Group×Time	*F* = 32.068; *p* < 0.001; partial *η*^2^ = 0.559

PFME, pelvic floor muscle exercise; PMR, progressive muscle relaxation exercise; SD, standard deviation; Mixed ANOVA, mixed-design analysis of variance; partial *η*^2^, partial eta squared.

*UIFCQ scores are presented as mean item scores (range: 1–6), calculated by dividing the total questionnaire score by the number of items (28), to maintain a standardized metric across dimensions.

The main effect of group was not statistically significant (F = 0.891, *p* = 0.450, partial *η*^2^= 0.034), indicating that the overall mean UIFCQ scores did not differ significantly among the four study groups when all measurement occasions were considered together.

In contrast, a significant main effect of time was observed (F = 43.611, *p* < 0.001, partial *η*^2^= 0.365), indicating that participants' comfort scores changed significantly over the study period. Bonferroni-adjusted pairwise comparisons demonstrated significant differences between baseline and Weeks 2, 4, and 6 (all *p* < 0.001). A significant difference was also found between Weeks 2 and 6 (*p* < 0.001). However, no significant differences were observed between Weeks 2 and 4 (*p* = 0.270) or between Weeks 4 and 6 (*p* = 0.080).

The group×time interaction, which represented the primary finding of the study, was statistically significant (F = 32.068, *p* < 0.001, partial *η*^2^ = 0.559). This significant interaction indicated that changes in comfort scores over time differed across the four study groups. The magnitude and timing of improvement were not uniform across the intervention groups. The PFME group demonstrated an early improvement that was largely maintained thereafter, whereas the PMR group showed a more progressive increase in comfort scores across the follow-up period. In the combined PFME + PMR group, improvement became more evident from Week 4 onward. In contrast, the control group showed a slight decline in comfort scores from baseline, followed by relative stabilization. The partial eta-squared value indicated a large interaction effect. Therefore, follow-up simple-effects analyses were conducted to further characterize the significant group×time interaction.

To further explore the significant interaction, Bonferroni-adjusted simple effects analyses were performed. In the PFME group, significant improvements in comfort scores were observed between baseline and Weeks 2 (*p* = 0.002), 4 (*p* < 0.001), and 6 (*p* < 0.001), whereas no significant differences were found among the follow-up assessments (all *p* > 0.05), indicating that the initial improvement was maintained throughout the intervention period.

In the PMR group, significant improvements were observed between baseline and all subsequent assessments (all *p* < 0.001). In addition, significant differences were identified between Weeks 2 and 4 and between Weeks 2 and 6 (both *p* < 0.001), whereas no significant difference was found between Weeks 4 and 6 (*p* > 0.05). These findings indicate that improvements continued until the later stages of follow-up.

In the combined PFME + PMR group, no significant difference was observed between baseline and Week 2 (*p* = 0.085). However, significant improvements were found between baseline and Week 4 (*p* = 0.013) and between baseline and Week 6 (*p* < 0.001). A significant difference was also observed between Weeks 2 and 6 (*p* = 0.006), whereas all remaining pairwise comparisons were not statistically significant (all *p* > 0.05). These findings suggest that improvements in comfort became more evident during the later follow-up assessments.

In the control group, significant differences were observed between baseline and Weeks 2 (*p* = 0.002), 4 (*p* < 0.001), and 6 (*p* < 0.001). A statistically significant, although relatively small, difference was also identified between Weeks 2 and 4 (*p* = 0.049). No significant differences were observed between Weeks 2 and 6 or between Weeks 4 and 6 (both *p* > 0.05).

Taken together, the significant group×time interaction demonstrated that the temporal pattern of change in comfort scores differed across the study groups. The PFME intervention was associated with an early improvement that was maintained throughout follow-up, while the PMR intervention produced a more progressive improvement, with further gains observed between Weeks 2 and 4 and between Weeks 2 and 6. The combined PFME + PMR intervention showed a delayed pattern of improvement, with statistically significant gains becoming evident from Week 4. In contrast, the control group exhibited a modest decline in comfort scores after baseline, followed by relative stabilization. These findings indicate that the interventions were associated with distinct trajectories of change in comfort rather than a uniform change over time.

## 4 Discussion

In the present study, participants' Urinary Incontinence and Frequency Comfort Questionnaire (UIFCQ) scores were assessed at four different time points. Although no significant differences were observed among the study groups at baseline, the interventions significantly influenced the pattern of change in comfort scores over time. Importantly, however, the statistically significant group×time interaction should be interpreted in conjunction with the magnitude of the observed changes. The absolute changes in UIFCQ scores were relatively small, and therefore, statistical significance should not be equated directly with clinical significance. These findings suggest that the effectiveness of the interventions should be interpreted not only in terms of overall group differences but also by considering the longitudinal trajectories of comfort throughout the follow-up period.

Conservative interventions, particularly PFME and relaxation-based approaches, are widely recommended as first-line treatment options for urinary incontinence, with their therapeutic benefits generally becoming evident over time. A multicenter randomized controlled trial conducted by Hagen et al. (2020) demonstrated that PFME significantly reduced urinary incontinence symptoms and improved quality of life in women with urinary incontinence (2). Consistent with these findings, the progressive increase in comfort scores observed during follow-up in the present study suggests a favorable change in perceived comfort over time following pelvic floor-focused interventions. Nevertheless, because the present study did not establish a minimal clinically important difference for the UIFCQ, the observed increases should be considered evidence of a favorable change in comfort rather than definitive evidence of a clinically important improvement.

In the present study, participants in the PFME group demonstrated significant improvements in comfort scores at Weeks 2, 4, and 6 compared with baseline, whereas no additional significant differences were observed among the follow-up assessments. This pattern indicates that PFME was characterized primarily by an early improvement followed by stabilization rather than continuous incremental gains. Mechanistically, PFME primarily targets pelvic floor muscle strength, endurance, and functional support of the urethra, which may explain the relatively rapid improvement observed during the early weeks of the intervention. These findings suggest that pelvic floor muscle exercise provides early functional benefits and that these improvements can be maintained throughout the intervention period with continued adherence to the exercise program. Previous studies have reported that the therapeutic effects of PFME generally become evident during the first weeks or months of treatment, while the long-term maintenance of these benefits largely depends on sustained exercise adherence. In a randomized controlled trial, Hagen et al. (2020) reported significant improvements in urinary incontinence symptoms among women receiving PFME; however, the addition of adjunctive interventions provided only limited benefit beyond routine PFME (2). These findings are consistent with the pattern observed in the present study, in which early improvements in the PFME group were maintained without substantial additional gains during subsequent follow-up.

In contrast, participants in the PMR group exhibited a more gradual pattern of improvement, with significant changes continuing until the later follow-up assessments. Unlike PFME, which primarily emphasizes muscle strengthening, PMR is intended to reduce excessive muscle tension and improve the ability to voluntarily relax the pelvic floor. Thus, the temporal pattern observed in the PMR group may reflect a more gradual adaptation in muscle relaxation, awareness, coordination, and neuromuscular control rather than an immediate strengthening effect. These findings suggest that, in addition to strengthening the pelvic floor muscles, improving muscle relaxation, coordination, and neuromuscular control may contribute to better urinary function. Relaxation-based interventions may be particularly beneficial for women with excessive pelvic floor muscle activity or dysfunctional muscle contraction patterns (22). Furthermore, current evidence emphasizes the importance of individualized approaches in pelvic floor rehabilitation. In a randomized controlled trial, Figueiredo et al. (2020) compared individual, group-based, and progressive group training programs for women with stress urinary incontinence and demonstrated that different training models produced significant symptom improvement over time (14). These findings may help explain the distinct temporal response patterns observed among the intervention groups in the present study. Accordingly, the later improvement observed with PMR should not necessarily be interpreted as evidence that PMR is superior to PFME; rather, the two interventions may influence different components of pelvic floor function and may therefore produce different temporal trajectories of change.

In the combined PFME + PMR group, significant improvements were observed at Weeks 4 and 6, suggesting that the combined intervention may produce a more gradual yet progressive therapeutic effect. This temporal pattern differed from both PFME and PMR alone: compared with the early response observed with PFME and the more gradual response observed with PMR, the combined intervention demonstrated significant improvement only from Week 4 onward. This may reflect the simultaneous targeting of complementary mechanisms—muscle strengthening and endurance through PFME, together with relaxation, coordination, and neuromuscular control through PMR. Because combined rehabilitation strategies simultaneously target multiple mechanisms, including muscle strength, endurance, relaxation capacity, and neuromuscular coordination, a longer period may be required for their full clinical benefits to become evident. Recent studies have similarly reported that combining pelvic floor muscle training with complementary exercise approaches may enhance symptom control and improve quality of life. For example, randomized controlled trials published in 2024 demonstrated that combining pelvic floor muscle training with Pilates-based exercise resulted in significant improvements in urinary incontinence symptoms and pelvic floor function (23, 24). Although these interventions differ from progressive muscle relaxation, they support the concept that multimodal conservative rehabilitation may provide additional clinical benefits beyond pelvic floor muscle training alone. However, the present findings do not demonstrate that the combined intervention produced a clinically superior improvement in comfort compared with either intervention alone. Rather, they suggest that combining strengthening and relaxation components may be associated with a distinct, later-emerging pattern of change that warrants further investigation in larger and longer trials.

A finding of the present study was the pattern observed over time in the control group, which exhibited a slight decrease in comfort scores. In contrast to the intervention groups that demonstrated progressive gains in comfort, this slight decline in the control group further underscores the significant group×time interaction identified in the mixed-design ANOVA. Furthermore, the large partial eta-squared value (partial *η*^2^= 0.559) indicates that the interaction between group and time had a substantial effect size, suggesting that the interventions made a meaningful contribution to changes in comfort over the follow-up period. Nevertheless, the large statistical effect size should also be interpreted cautiously because effect size reflects the magnitude of the statistical interaction rather than the patient's perception of clinically meaningful benefit. Given the relatively small absolute changes in UIFCQ scores, the clinical relevance of this effect cannot be determined definitively.

These findings may support the integration of pelvic floor muscle training combined with relaxation-based interventions into routine conservative management programs for women with stress urinary incontinence. From a mechanistic perspective, PFME may be particularly relevant when the primary therapeutic goal is improving pelvic floor strength and functional support, whereas PMR may be more appropriate when relaxation and neuromuscular coordination are important treatment targets. A combined approach may be considered when both strengthening and relaxation are desired; however, the present findings do not provide sufficient evidence to conclude that the combined intervention is clinically superior to PFME or PMR alone. Incorporating relaxation techniques alongside strengthening exercises may improve patient comfort and adherence to treatment. In addition to urinary symptoms, pelvic dysfunctions and radical interventions are well known to significantly impair female sexual function and overall quality of life, further underscoring the necessity of patient-centered evaluation in routine care (25).

Although the group×time interaction was statistically significant, the absolute changes in mean UIFCQ scores were relatively small across the six-week follow-up period. Therefore, the statistical significance observed in the present study should not necessarily be interpreted as evidence of a clinically meaningful improvement. The relatively high baseline comfort scores may have limited the magnitude of observable change and may have contributed to a ceiling effect. In addition, a minimal clinically important difference for the UIFCQ has not been established, to our knowledge, which limits the ability to determine whether the observed changes are clinically meaningful from the participants' perspective. Thus, the present findings should be interpreted as demonstrating statistically detectable changes in the trajectory of comfort rather than establishing a clinically important magnitude of improvement. The distinction is particularly important because the UIFCQ captures perceived comfort, and small numerical changes may not necessarily correspond to noticeable improvements in daily life. Future studies with larger samples, longer follow-up periods, and an established minimal clinically important difference or other patient-centered thresholds for the UIFCQ are warranted to further evaluate the clinical relevance of these findings.

## 5 Strengths and limitations

The primary strength of this study is its randomized controlled design, which enabled the evaluation of the effects of PFME, PMR, and their combined application on comfort among women with stress urinary incontinence. Assessing outcomes at four different time points allowed not only the determination of intervention effectiveness but also the characterization of longitudinal trajectories of comfort over time. Furthermore, comparing PFME, PMR, and their combined application within a single study provides valuable evidence regarding the relative effectiveness of different conservative treatment strategies. The use of standardized intervention protocols, a validated outcome measure, and the reporting of effect sizes further enhanced the internal validity and clinical interpretation of the findings.

This study also has several limitations. First, the single-center design and relatively small sample size may limit the generalizability of the findings to broader populations. Second, because the intervention period was limited to six weeks, long-term sustainability and effectiveness could not be evaluated. Third, participants were allocated to intervention groups based on clinic days to minimize cross-contamination in the outpatient setting. As this approach did not involve individual randomization, the possibility of selection bias cannot be excluded. Fourth, due to post-randomization dropouts, analyses were conducted on a Per-Protocol basis rather than an Intent-to-Treat approach, which may introduce attrition bias. Fifth, assessing exercise adherence and follow-up outcomes exclusively through participant self-reports and telephone interviews-without objective tools (e.g., perineometry, EMG) or direct verification-may have introduced self-report and social desirability biases, as participants might overestimate their compliance or comfort levels. Future multicenter trials with larger sample sizes, longer follow-up, individual randomization, and objective functional outcomes are warranted to confirm these findings.

## 6 Conclusions

This study evaluated longitudinal changes in comfort among women with stress urinary incontinence receiving PFME, PMR, combined PFME + PMR, or standard care. Although no significant overall group effect was observed, significant time and group×time effects indicated that intervention-related improvements emerged over time and followed different trajectories across groups. PFME produced early and sustained improvements, PMR showed progressive benefits over time, and combined PFME + PMR demonstrated significant improvements during later follow-up assessments. These findings suggest that both PFME and PMR are effective conservative interventions for improving comfort in women with stress urinary incontinence. Individualized pelvic floor rehabilitation programs incorporating muscle strengthening, relaxation, and neuromuscular coordination may optimize patient outcomes. Further multicenter studies with larger samples and longer follow-up are warranted to confirm these findings.

## Data Availability

The original contributions presented in the study are included in the article/Supplementary Material, further inquiries can be directed to the corresponding author/s.

## References

[1] International Continence Society. Stress urinary incontinence. Available online at: https://www.ics.org/committees/standardisation/terminologydiscussions/sui (Accessed July 15, 2026).

[2] Hagen S Elders A Stratton S Sergenson N Bugge C Dean S Hay-Smith J Kilonzo M Dimitrova M Abdel-Fattah M Agur W Booth J Glazener C Guerrero K McDonald A Norrie J Williams LR McClurg D, Effectiveness of pelvic floor muscle training with and without electromyographic biofeedback for urinary incontinence in women: multicentre randomised controlled trial. Br Med J*.* (2020) 371:m3719. 10.1136/bmj.m3719 PMC7555069 33055247

[3] Cardozo L Rovner ES Wagg A Wein AJ Abrams P. Incontinence; 7th ed. International Consultation on Incontinence–International Continence Society: Bristol: International Continence Society (ICI-ICS) (2023).

[4] Milsom I Altman D Cartwright R Lapitan MC Nelson R Sjöström S. Epidemiology of urinary incontinence (UI) and other lower urinary tract symptoms (LUTS), pelvic organ prolapse (POP), and anal incontinence (AI). In: Cardozo L Rovner ES Wagg A Wein AJ Abrams P, Eds. Incontinence; 6th ed., Bristol: International Continence Society (2017) pp. 1–141.

[5] Partin AW Dmochowski RR Kavoussi LR Peters CA Wein AJ, Eds. *Campbell-Walsh-Wein Urology*; 12th ed., Philadelphia, PA: Elsevier (2021).

[6] European Association of Urology. *EAU Guidelines on Non-Neurogenic Female Lower Urinary Tract Symptoms*; EAU Guidelines Office, (2025). Available online at: https://uroweb.org/guidelines (Accessed July 15, 2026).

[7] Kaya S Akbayrak T Gürşen C Beksaç S. Short-term effect of adding pelvic floor muscle training to bladder training for female urinary incontinence: a randomized controlled trial. Int Urogynecol J*.* (2015) 26:285–93. 10.1007/s00192-014-2517-4 25266357

[8] Kolcaba K. Comfort Theory and Practice: A Vision for Holistic Health Care and Research. New York, NY: Springer Publishing Company (2003).

[9] Dowd T Kolcaba KY Steiner R. Using cognitive strategies to enhance bladder control and comfort. Holist Nurs Pract. (2000) 14:91–103. 10.1097/00004650-200001000-00013 12119974

[10] Zengin N. İdrar Kaçıran Kadınlarda Hemşirelik Eğitimi ve Davranışsal Tedavinin Konfor, Pelvik Taban Kas Egzersizi Uygulaması, Öz-Etkililik Algısı ve Yaşam Kalitesine Etkisi (Thesis No. 224600) [Doctoral Dissertation]. Istanbul, Türkiye: Marmara University (2008).

[11] Hakbilen HG Ince S. Comfort theory-based nursing ınterventions in women with stress urinary incontinence: randomized controlled trial. Int Urogynecol J. (2026):1–16. 10.1007/s00192-026-06735-z 42301318

[12] Zaccardi JE Wilson L Mokrzycki ML. The effect of pelvic floor re-education on comfort in women having surgery for stress urinary incontinence. Urol Nurs. (2010) 30(2):148. 10.7257/1053-816X.2010.30.2.137 20469573

[13] Luginbuehl H Lehmann C Koenig I Kuhn A Buergin R Radlinger L. Involuntary reflexive pelvic floor muscle training in addition to standard training versus standard training alone for women with stress urinary incontinence: a randomized controlled trial. Int Urogynecol J. (2022) 33:531–40. 10.1007/s00192-021-04701-5 33566173 PMC8885550

[14] Figueiredo VB Nascimento SL Martínez RFL Lima CTS Ferreira CHJ Driusso P. Effects of individual pelvic floor muscle training vs individual training progressing to group training vs group training alone in women with stress urinary incontinence: a randomized clinical trial. Neurourol Urodyn*.* (2020) 39:1447–55. 10.1002/nau.24370 32353206

[15] Wang X Qiu J Li D Wang Z Yang Y Fan G. Pressure-mediated biofeedback with pelvic floor muscle training for urinary incontinence: a randomized clinical trial. JAMA Netw Open. (2024) 7:e2442925. 10.1001/jamanetworkopen.2024.42925 39499517 PMC11539013

[16] Woodley SJ Lawrenson P Boyle R Cody JD Mørkved S Kernohan A. Pelvic floor muscle training for preventing and treating urinary and faecal incontinence in antenatal and postnatal women. Cochrane Database Syst Rev. (2020) 5:CD007471. 10.1002/14651858.CD007471.pub4 32378735 PMC7203602

[17] Zendehdel M Elyasi F Jahanfar S Emami-Sahebi A. Effectiveness of progressive muscle relaxation technique on anxiety caused by COVID-19 in pregnant women: a randomized clinical trial. Neuropsychopharmacol Rep*.* (2022) 42:158–65. 10.1002/npr2.12241 35182048 PMC9216373

[18] Toqan D Ayed A Khalaf IA. Effect of progressive muscle relaxation exercise on anxiety among nursing students prior to critical care clinical training. SAGE Open Nurs*.* (2023) 9:22799036231181668. 10.1177/22799036231181668 PMC10272653 37333031

[19] Schulz KF Altman DG Moher D, CONSORT Group. CONSORT 2010 statement: updated guidelines for reporting parallel group randomised trials. Br Med J. 2010 340:c332. 10.1136/bmj.c332 PMC2844940 20332509

[20] Faul F Erdfelder E Lang AG Buchner A. G*power 3: a flexible statistical power analysis program for the social, behavioral, and biomedical sciences. Behav Res Methods. (2007) 39(2):175–91. 10.3758/bf03193146 17695343

[21] Tabachnick BG Fidell LS. Using Multivariate Statistics. 6th ed. Boston, MA: Pearson; (2013).

[22] Dumoulin C Cacciari LP Hay-Smith EJC. Pelvic floor muscle training versus no treatment, or inactive control treatments, for urinary incontinence in women. Cochrane Database Syst Rev*.* (2018) 10:CD005654. 10.1002/14651858.CD005654.pub4 30288727 PMC6516955

[23] Dimli BO Mutlu EK Altaç DS Taşkıran H. Comparison of the effects of pelvic floor muscle training and modified pilates exercises in elderly women with stress urinary incontinence: a randomized clinical trial. Eur J Obstet Gynecol Reprod Biol*.* (2024) 300:327–36. 10.1016/j.ejogrb.2024.07.033 39084033

[24] Gonzaga S De Oliveira RG Dutra LL Oliveira LS De Oliveira LC. Comparative analysis of pelvic floor muscle training and pilates in managing urinary incontinence among postmenopausal women: a randomized controlled trial. Int Urogynecol J*.* (2024) 35:561–9. 10.1007/s00192-023-05712-0 38206341

[25] Bizzarri FP Campetella M Recupero SM Bellavia F D'Acco L Rossi F. Female sexual function after radical treatment for MIBC: a systematic review. Journal Personalized Medicine. (2025) 15(9):415. 10.3390/jpm15090415 PMC12471525 41003117

